# Vitamin D and Gestational Diabetes Mellitus in the IEU OpenGWAS Project: A Two-Sample Bidirectional Mendelian Randomization Study

**DOI:** 10.3390/nu16172836

**Published:** 2024-08-24

**Authors:** Yuxuan Bai, Xiaoxiao Wang, Yaxuan Xu, Chang Jiang, Haoran Liu, Zixiu Xu, Jinping Shen, Xumei Zhang, Qiang Zhang, Yue Du

**Affiliations:** 1 Department of Occupational and Environmental Health, School of Public Health, Tianjin Medical University, Tianjin 300070, China; baiyuxuan@tmu.edu.cn (Y.B.); wangxiaoxiao@tmu.edu.cn (X.W.); jiangchang@tmu.edu.cn (C.J.); liuhaoran@tmu.edu.cn (H.L.); shenjinping@tmu.edu.cn (J.S.); qiangzhang@tmu.edu.cn (Q.Z.); 2 Key Laboratory of Prevention and Control of Major Diseases in the Population, Ministry of Education, Tianjin Medical University, Tianjin 300070, China; xuyaxuan@tmu.edu.cn (Y.X.); xuzixiu@tmu.edu.cn (Z.X.); zhangxumei@tmu.edu.cn (X.Z.); 3 School of Nursing, Tianjin Medical University, Tianjin 300070, China; 4 Department of Nutrition and Food Science, School of Public Health, Tianjin Medical University, Tianjin 300070, China; 5 Department of Health Management, School of Public Health, Tianjin Medical University, Tianjin 300070, China

**Keywords:** vitamin D, gestational diabetes mellitus, Mendelian randomization

## Abstract

Background: Gestational diabetes mellitus (GDM) is one of the most prevalent pregnancy problems, and there is still debate over the relationship between vitamin D and GDM. Objectives: Our objective is to investigate the correlation between vitamin D and GDM by employing Mendelian randomization (MR) with summary data obtained from genome-wide association studies (GWAS). Methods: Data on exposures and outcomes, namely vitamin D, vitamin D insufficiency, and GDM, were acquired from the IEU OpenGWAS Project. Bidirectional MR analysis was performed utilizing the inverse variance weighted (IVW) method as the principal analytical approach. The complementary approaches employed in this study encompassed weighted median, simple mode, weighted mode, and MR-Egger regression. A series of sensitivity analysis were conducted in order to assess the reliability of the obtained results. Results: The data were acquired from the IEU OpenGWAS Project. Following the application of the three assumptions of MR, 13 single nucleotide polymorphisms (SNPs) were included in the MR analysis for vitamin D levels and vitamin D deficiency on GDM, and 10 and 26 SNPs were included for GDM on vitamin D levels and deficiency, respectively. The findings from the IVW analysis revealed a significant positive correlation between vitamin D levels and GDM (OR = 1.057, 95% CI: 1.011–1.104, *p* = 0.015). Conversely, a negative correlation was seen between vitamin D deficiency and GDM (OR = 0.979, 95% CI: 0.959–0.999, *p* = 0.039). The results of the reverse MR study revealed no evidence of reverse causation between GDM and vitamin D. The findings from multiple MR approaches were in line with the direction of IVW analysis. Sensitivity analysis revealed no evidence of heterogeneity, pleiotropy, or outliers, suggesting the robustness of the results. Conclusions: There exists a causal association between vitamin D and GDM, whereby vitamin D levels serve as a risk factor for GDM.

## 1. Introduction

Vitamin D is a crucial micronutrient in the human body that is soluble in fat and plays a critical role in the metabolism of calcium and phosphorus. Besides enhancing the absorption of calcium, magnesium, and phosphate in the intestines, it has multiple biological effects [1,2]. For instance, vitamin D regulates nerve and muscle function, reduces inflammation, influences gene transcription and translation, and regulates various cells’ proliferation, differentiation, and apoptosis [3]. It has the potential to exert a positive influence on health across various dimensions, encompassing the preservation of bone health, mitigation of all-cause mortality, and reduction of fall-related risks [4]. Thus, the importance of vitamin D is indisputable. When the body lacks sufficient vitamin D due to inadequate dietary intake or insufficient sun exposure, it can lead to vitamin D deficiency or insufficiency [5]. A retrospective study conducted by Palacios et al. revealed that there exists a prevalent nutritional epidemic of vitamin D insufficiency among pregnant women on a global scale [6]. The study conducted by Bandeira et al. demonstrated that a shortage of vitamin D might result in unfavorable pregnancy outcomes and exert enduring impacts on the well-being of both the maternal and fetal health [7]. Numerous studies have provided evidence suggesting that pregnant women who experience a shortage of vitamin D face an elevated likelihood of developing gestational diabetes, gestational hypertension, preeclampsia, anemia, and various other unfavorable consequences during pregnancy. The aforementioned consequences encompass diminished fertility, heightened disease activity, placental dysfunction, and the birth of infants small for gestational age [8,9].

Gestational diabetes mellitus (GDM) is characterized by differential glucose tolerance of varying degrees that arises or is initially identified during pregnancy. It falls under the classification of diabetes mellitus diagnosed between the 24th and 28th week of gestation but is distinct from type 1 or type 2 diabetes mellitus diagnoses [10,11]. Recent research has indicated an upward trend in the prevalence of GDM, with worldwide rates ranging from 15% to 20%, making it an increasing risk factor for more and more adverse pregnancy outcomes. A body of compelling evidence suggests a correlation between GDM and many adverse outcomes, including immediate complications for both the mother and newborn, as well as long-term health implications [12,13,14]. Therefore, actively identifying and investigating the risk factors for GDM and proactively focusing on prevention and early intervention are crucial for addressing the increasing prevalence of GDM and alleviating the resulting burden of the condition [15]. Recent research indicates that vitamin D could potentially play a role in the development of GDM [16]. This underscores the need to investigate the effect of vitamin D in pregnancy in order to enhance results for both maternal and offspring health.

Current international epidemiological studies on vitamin D, vitamin D deficiency, and GDM yield inconsistent conclusions. Dragomir et al. reported an association between vitamin D deficiency and gestational diabetes in a retrospective study [17]. Additionally, Wang et al. reported in a meta-analysis that vitamin D deficiency is closely associated with an increased risk of GDM [18]. Research indicates a correlation between low vitamin D levels in the maternal serum and GDM. Case-control studies conducted in Turkey and China have documented that individuals diagnosed with GDM have comparatively lower mean serum vitamin D levels in comparison to pregnant women without GDM [19,20]. Nevertheless, alternative research has produced no substantial correlation between vitamin D levels and GDM. Two case-control studies conducted in Europe found no statistically significant disparity in the mean serum vitamin D levels comparing patients with GDM and pregnant women [21,22]. A cross-sectional study from the Philippines indicated that GDM patients had higher average serum vitamin D levels than the normal pregnant population [16]. As a result of the requirement for more agreement among diverse epidemiological investigations, additional investigation is imperative to clarify the causal association between vitamin D and GDM. This will provide insights into the mechanisms involved and evidence for clinical diagnosis and treatment.

Mendelian randomization (MR) provides a novel method for causal inference in epidemiology using genotype as an instrumental variable. The efficacy of this approach is contingent upon three fundamental assumptions: a strong association between the instrumental variable and the exposure factor, the absence of any correlation between the instrumental variable and confounding factors, and the indirect influence of the instrumental variable on the outcome solely through the exposure factor. The utilization of these principles in MR facilitates the inference of the association between exposure and outcome without the influence of confounding variables and reverse causality. It has been widely applied in numerous studies [23,24]. This study uses publicly accessible summary data from genome-wide association studies (GWAS) to investigate the causal connection between vitamin D and GDM using MR. The objective is to offer more scientific evidence for risk assessment in maternal and infant health.

## 2. Materials and Methods

**Figure 1:**
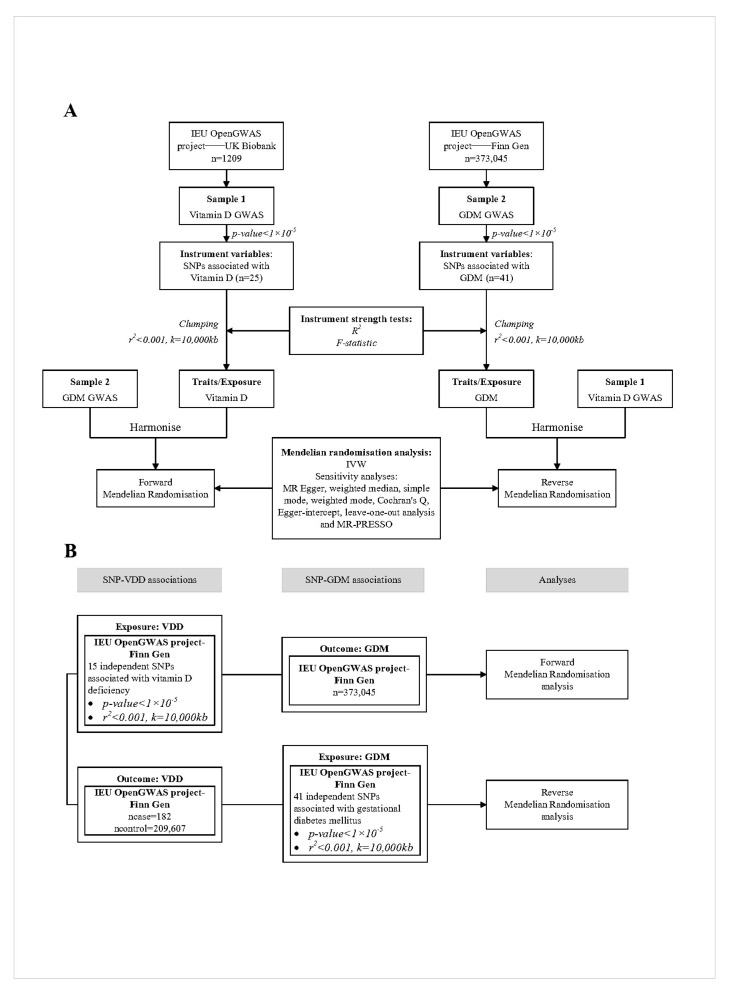
Overview of the two-sample MR study design used to investigate the probability of bidirectional association between vitamin D and GDM. (**A**). Bidirectional MR study of vitamin D and GDM. (**B**). Bidirectional MR study of vitamin D deficiency and GDM. The results of (**B**) are used to validate the results of (**A**). VDD, vitamin D deficiency; GDM, gestational diabetes mellitus; GWAS, genome-wide association study; IVW, inverse-variance weighted; MR, Mendelian randomization; SNP, single nucleotide polymorphism.

A summary of the two-sample MR study design for this study is shown below in Figure 1.

### 2.1. Study Design and Data Sources

The present study conforms to the recently launched STROBE-MR criteria for the reporting of MR investigations [25]. In order to evaluate the influence of vitamin D levels on GDM, we employed single nucleotide polymorphisms (SNPs) linked to vitamin D levels from the UK Biobank database as instrumental variables. To further validate the relationship, we also assessed the effect of vitamin D deficiency on GDM using SNPs associated with vitamin D deficiency in the FinnGen database as instrumental variables. Additionally, to evaluate the impact of GDM on vitamin D levels and deficiency, we used SNPs associated with GDM from the FinnGen database as instrumental variables.

#### 2.1.1. Principles of SNP Selection

**Figure 2:**
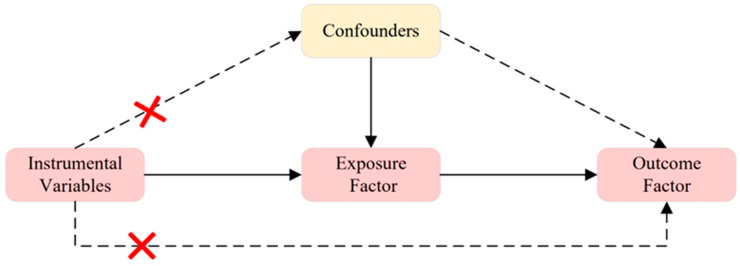
Three Assumptions of MR. Assumption 1: The instrumental variable is closely related to the exposure factor; Assumption 2: The instrumental variable is not influenced by known or unknown confounding factors; Assumption 3: The instrumental variable affects the outcome factor only through the exposure factor. A red cross indicates that exposure does not affect outcome in this pathway.

As shown in Figure 2, three conditions must be met for instrumental variables in MR analysis: (1) relevance assumption: the instrumental variable must be closely related to the exposure factor; (2) independence assumption: the instrumental variable should not be affected by known or unknown confounding factors; (3) exclusion restriction: the instrumental variable affects the outcome factor only through the exposure factor. To identify qualified instrumental variables that meet three core MR assumptions [26], we employed a series of quality control methods. We selected SNPs with a genome-wide significance threshold of *p* < 5 × 10^−8^ that are closely related to the exposure factor [27]. However, using this threshold, only a few SNPs were identified as instrumental variables for “Vitamin D deficiency”, “Vitamin D”, and “GDM”. Therefore, we applied a second threshold, selecting SNPs with a genome-wide significance threshold of *p* < 1 × 10^−5^ to uncover more potential causal relationships between exposure and outcomes [26,28]. We then filtered out SNPs associated with confounding factors and outcomes with r^2^ > 0.001 to avoid linkage disequilibrium within a 10,000 kb range. Potential confounding factors associated with the selected SNPs were analyzed using the PhenScannerV2 database [http://www.phenoscanner.medschl.cam.ac.uk/, accessed on 4 February 2024], and SNPs with phenotypes significantly related to GDM or that could alter outcomes were excluded. Additionally, SNPs that had a significant impact on the entire data set during the leave-one-out analysis were excluded from the initial MR analysis.

#### 2.1.2. Data Sources for Vitamin D Levels and SNP Selection 

The GWAS summary statistics for 15,533,459 SNPs associated with vitamin D levels were obtained from the IEU OpenGWAS Project. This database, created by the MRC Integrative Epidemiology Unit (IEU) at the University of Bristol, is a manually curated compilation of comprehensive GWAS summary datasets. It can be accessed either by downloading or by making queries (https://gwas.mrcieu.ac.uk/, accessed on 4 February 2024).

Our study focused on the UK Biobank data within the IEU OpenGWAS Project, which provides the largest and most recent GWAS data for vitamin D levels (GWAS ID: ukb-e-100021_AFR). The UK Biobank is a prospective cohort research that involved the recruitment of more than 500,000 people within the age range of 40 to 69 in the United Kingdom from 2006 to 2010 [29]. The participants in this study underwent genotyping and were subjected to long-term clinical follow-up [29].

SNPs that are linked to vitamin D levels were chosen for the purpose of our analysis. After exclusions, our study included 13 SNPs (see Supplementary Materials, Table S2) used as genetic instruments to predict vitamin D deficiency from a genetic perspective. Based on the genome-wide threshold (*p* < 1 × 10^−5^), 25 SNPs associated with vitamin D deficiency were selected through linkage disequilibrium (LD)-based pruning. After screening and removing SNPs with significant influence in the initial MR analysis and excluding some SNPs during data extraction for GDM, 13 SNPs were finally included in the final MR analysis.

#### 2.1.3. Data Sources for Gestational Diabetes Mellitus and SNP Selection

The GWAS summary statistics for 16,379,684 SNPs associated with GDM were obtained from the IEU OpenGWAS Project (https://gwas.mrcieu.ac.uk/, accessed on 4 February 2024). Our study focused on the FinnGen study data within this project, which provides the largest and most recent GWAS data for GDM (GWAS ID: finn-b-O15_PREG_DM). The dataset encompasses genetic information sourced from the FinnGen biobank, as well as related national health registry data derived from a total of 12,859 cases and 73,341 controls. The researchers at FinnGen employed a range of Illumina and Affymetrix GWAS chips to genotype the cohort of people. Genotype interpolation was performed using the Beagle v.4.1 program, employing a population-specific reference panel consisting of 3775 high-coverage whole genome sequences originating from Finland. In the reverse MR analysis, we selected SNPs associated with GDM. Following exclusions, our analysis included 26 SNPs for vitamin D deficiency and 10 SNPs for vitamin D levels as different outcomes (see the Supplementary Materials, Tables S3 and S4). These SNPs were used as genetic instruments to predict GDM from a genetic perspective. Based on the genome-wide threshold (*p* < 1 × 10^−5^), 41 SNPs associated with GDM were selected through LD-based pruning. After screening and removing SNPs using the PhenScannerV2 database, considering minor allele frequency, initial MR analysis leave-one-out results, culling palindromic sequences, and information loss during data extraction for vitamin D deficiency and vitamin D levels, 26 SNPs and 10 SNPs were finally included in the final MR analysis.

#### 2.1.4. Data Sources for Vitamin D Deficiency and SNP Selection

The GWAS summary statistics for 16,380,446 SNPs associated with vitamin D deficiency were obtained from the IEU OpenGWAS Project (https://gwas.mrcieu.ac.uk/, accessed on 4 February 2024). Our study focused on the FinnGen study data within the IEU OPEN GWAS PROJECT (GWAS ID: finn-b-E4_VIT_D_DEF).

We selected SNPs strongly associated with vitamin D deficiency for the analysis. After exclusions, our study included 13 SNPs (see Supplementary Materials, Table S1) used as genetic instruments to predict vitamin D deficiency from a genetic perspective. Based on the genome-wide threshold (*p* < 1 × 10^−5^), 15 SNPs associated with vitamin D deficiency were selected through LD-based pruning. After considering minor allele frequency, the PhenScannerV2 database, and initial MR analysis leave-one-out results, 13 SNPs were ultimately selected for the final MR analysis.

### 2.2. Statistical Power (R^2^ and F Value)

In MR analysis, the F statistic measures the strength of the instrumental variable (IV) for the associated exposure [30]. The F statistic is calculated using the formula [(n − k − 1)/k] × [R^2^/(1 − R^2^)], where R^2^ represents the variance in exposure explained by the genetic instrument, k represents the number of genetic variants, and n represents the sample size. R^2^ is calculated using the formula (−2) × β^2^ × EAF × (1 − EAF)/2 × β^2^ × EAF × (1 − EAF) + se^2^ × 2 × N × EAF (1 − EAF). Here, EAF represents the effect of allele frequency. Based on the exposure data, we calculated the F statistic for each instrument to verify its strength and to estimate the effect of sample overlap and weak instrument bias. An F statistic greater than 10 is considered sufficient to mitigate potential bias. In contrast, instrumental variables with an F statistic less than 10 are considered weak instruments and are excluded from the MR analysis [31,32].

### 2.3. Mendelian Randomization Analysis

This study employed the inverse variance weighted (IVW) random effects method as the primary MR analysis technique. The IVW method, also known as the Toby Johnson method, provides causal estimates based on summary data from multiple instruments. It combines the Wald estimates of each genetic variant using an IVW meta-analysis approach, utilizing the variance from the first term of the delta method. It is important to note that the IVW method assumes all instrumental variables satisfy the MR assumptions and that no intercept term exists [33]. The analysis results may be inaccurate if the instrumental variables do not meet these assumptions.

### 2.4. Sensitivity Analysis

Sensitivity analyses were conducted using MR-Egger, weighted median, simple mode, and weighted mode methods [34,35,36]. These methods, with slightly different assumptions, provide the most substantial evidence for causal inference when results are consistent across multiple approaches [37].

Cochran’s Q test was performed on SNPs that met all assumptions using the mr_heterogeneity software package to assess heterogeneity among individual genetic variants [38,39]. A *p*-value less than 0.05 for Cochran’s Q test indicates heterogeneity, suggesting that different ages and genders influence the relationship between exposure and outcome. In the research process, the IVW fixed effects model is initially used, followed by a heterogeneity test. If heterogeneity is observed, the IVW random effects model is used for re-analysis, with its results considered the gold standard. If no evidence of heterogeneity is observed, the results of the IVW fixed-effects model are taken as the final results. Horizontal pleiotropy was assessed using the MR-Egger-intercept method and the MR-PRESSO test. For the MR-Egger-intercept method, a *p*-value less than 0.05 indicates the presence of horizontal pleiotropy, meaning that the genetic variants significantly influence the outcome through pathways other than exposure. Conversely, a *p*-value greater than 0.05 suggests that the exposure does not significantly influence the outcome through other pathways. The leave-one-out sensitivity test in the IVW analysis was used to sequentially exclude each SNP from the MR analysis to detect influential points [40]. The stability of the results was checked by observing the symmetry of the funnel plot. Additionally, outliers were identified using the MR-PRESSO method, and their impact on the results was assessed.

## 3. Results

### 3.1. Instrumental Variables

In exploring the causal relationship between vitamin D and GDM, data for 4 SNPs related to vitamin D levels and no SNPs related to vitamin D deficiency were missing during the extraction process for GDM. After culling palindromic sequences, we obtained 21 instrumental variables for vitamin D levels and 13 for vitamin D deficiency. Based on the criterion of excluding SNPs with an allele frequency < 0.01, two SNPs (rs77301237 and rs76932715) related to vitamin D deficiency were excluded. In the initial MR analysis, the leave-one-out analysis indicated that eight SNPs (rs7147651, rs6091005, rs1993507, rs62083469, rs2358324, rs11915910, rs11891159, and rs73250771) strongly associated with vitamin D levels had a significant impact on the overall dataset. After excluding these SNPs, we ultimately included 13 SNPs in the final MR analysis of vitamin D levels and GDM.

When exploring the causal relationship between GDM and vitamin D, data for 0 SNPs related to vitamin D deficiency and 13 SNPs related to vitamin D levels were missing during the extraction process. After culling palindromic sequences (rs6008117, rs7933420), we obtained 26 instrumental variables for vitamin D deficiency and 13 for vitamin D levels. Based on the criterion of excluding SNPs with an allele frequency < 0.01, six SNPs (rs137914808, rs75966627, rs138529718, rs141719793, rs151149684, rs73031073) related to GDM were excluded. Using the PhenoScanner database to search for related phenotypes, seven SNPs associated with vitamin D deficiency (rs1260326, rs7766070, rs9275373, rs2523504, rs13195441, rs7074440, rs10830963) were excluded, and another seven SNPs associated with vitamin D levels (rs1260326, rs7766070, rs9275373, rs2523504, rs13195441, rs7074440, rs10830963) were excluded. In the initial MR analysis exploring the relationship between GDM and vitamin D levels, the leave-one-out analysis indicated that three SNPs (rs10752161, rs59649116, rs111854973) strongly associated with GDM had a significant impact on the overall dataset. After excluding these SNPs, 10 instrumental variables were ultimately included in the final MR analysis of GDM and vitamin D levels. All selected SNPs met the instrumental variable selection criteria.

### 3.2. The Effect of Vitamin D on Gestational Diabetes Mellitus

**Figure 3:**
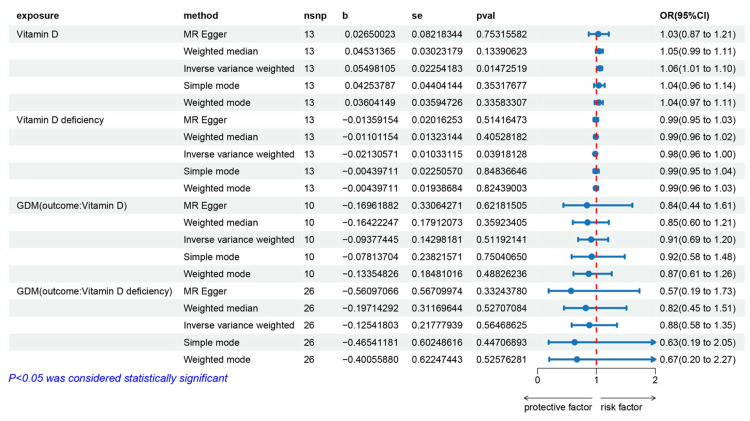
Forest plot of the MR analysis results for vitamin D levels, vitamin D deficiency, and their effects on gestational diabetes, as well as the impact of gestational diabetes on vitamin D levels and vitamin D deficiency. From top to bottom, the forest plot shows the results of the five methods of MR analysis on the relationships between (1) vitamin D levels and gestational diabetes, (2) vitamin D deficiency and gestational diabetes, (3) gestational diabetes and vitamin D levels, (4) gestational diabetes and vitamin D deficiency. Red dashed line: OR=1.

**Figure 4:**
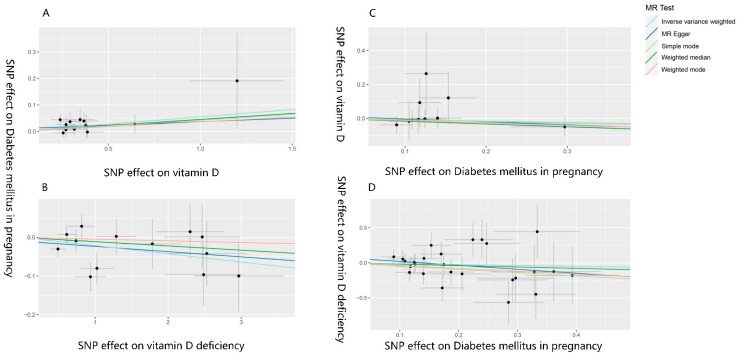
Scatter plot of the MR analysis results on vitamin D levels, vitamin D deficiency, and their effects on gestational diabetes, as well as the impact of gestational diabetes on vitamin D levels and vitamin D deficiency. (**A**–**D**) are scatter plots of the MR analysis results, showing the relationships between (**A**). vitamin D levels and GDM, (**B**). vitamin D deficiency and GDM, (**C**). GDM and vitamin D levels, and (**D**). GDM and vitamin D deficiency. A cross around each SNP show 95% CI. The slopes of each line represent the causal association for each method. MR, Mendelian randomization; SNP, single nucleotide polymorphism.

The IVW fixed effects model analysis indicated that genetically predicted vitamin D levels are a risk factor for GDM (OR = 1.057, 95% CI: 1.011–1.104, *p* = 0.015), demonstrating statistical significance (Figure 3 and Figure 4). Four additional methods were used to confirm the robustness of these results: MR-Egger, weighted median, simple mode, and weighted mode. Although these methods did not yield statistically significant results, their direction of effect was consistent with the IVW method (all OR values > 1, Figure 3 and Figure 4). Heterogeneity tests (see Supplementary Materials, Table S5) showed no evidence of heterogeneity (*p* > 0.05). Therefore, the aforementioned IVW fixed-effects model results are considered the final results. Horizontal pleiotropy tests (see Supplementary Materials, Table S5) also showed no evidence of horizontal pleiotropy. The leave-one-out sensitivity test (see Supplementary Materials, Figure S1) indicated that no single SNP significantly impacted the overall data. The funnel plot (see Supplementary Materials, Figure S2) exhibited good symmetry, suggesting stable and reliable results. Additionally, the analysis of genetically predicted vitamin D deficiency and GDM (OR = 0.979, 95% CI: 0.959–0.999, *p* = 0.039) further supported these findings.

### 3.3. The Effect of Gestational Diabetes Mellitus on Vitamin D

The IVW fixed effects model analysis showed no statistically significant association between genetically predicted GDM and vitamin D levels (OR = 0.910, 95% CI: 0.688–1.205, *p* = 0.512) or between GDM and vitamin D deficiency (OR = 0.882, 95% CI: 0.576–1.352, *p* = 0.565), indicating no evidence of reverse causality (Figure 3 and Figure 4). The heterogeneity test (see Supplementary Materials, Table S5) showed no heterogeneity (*p* > 0.05). Therefore, the aforementioned IVW fixed-effects model results are considered the final results. The horizontal pleiotropy test (see the Supplementary Materials, Table S6) also showed no evidence of horizontal pleiotropy. The leave-one-out sensitivity test (see Supplementary Materials, Figure S1) indicated that no single SNP significantly impacted the overall data, and the symmetry of the funnel plot (see Supplementary Materials, Figure S2) suggested that the results were stable and reliable.

## 4. Discussion

This study sought to examine the correlation between vitamin D levels and the occurrence of GDM, as well as to analyze the reverse causative relationship. The results indicate that higher vitamin D levels are associated with an increased probability of developing GDM, demonstrating a positive correlation. Conversely, vitamin D deficiency is negatively correlated with GDM. Potential confounding factors did not influence the process of inferring this causal relationship. Cochran’s Q and leave-one-out analysis identified no SNPs significantly impacting the results. Additionally, MR-Egger and MR-PRESSO tests did not detect any horizontal pleiotropy. Therefore, the sensitivity analysis and multiple comparisons correction confirm the reliability of these results.

The existing international epidemiological research findings provide conflicting evidence about the correlation between vitamin D and GDM in pregnant women. The study conducted by Lips et al. [41] revealed a negative correlation between vitamin D deficiency and blood glucose control. There have been findings suggesting that pregnant women with GDM have notably lower levels of vitamin D compared to pregnant women who are not diagnosed [21,41]. Mehdi et al. [42] employed a random effects model to incorporate a total of nine cohort studies and six nested case-control studies into their analysis. The linear analysis conducted shown that a rise of 10 nmol/L in serum 25-(OH)-D concentration was associated with a 2% reduction in the risk of GDM. However, some studies have shown no significant correlation between vitamin D and the incidence of GDM in pregnant women [43]. In their study, Park et al. [44] conducted an analysis on the serum vitamin D levels of a sample of 523 pregnant women from Korea during the gestational period of 12–14 weeks. Their findings indicated that there was no significant association between early maternal vitamin D insufficiency and GDM. Similarly, Boyle et al. [45], in a prospective cohort study of New Zealand women, found that although maternal vitamin D deficiency at 15 weeks of gestation was associated with GDM, this association was not significant after adjusting for body mass index and ethnicity. The authors suggest that inconsistencies in epidemiological studies may result from different regional environments, populations, and confounding factors. This study uses MR analysis to investigate the impact of vitamin D on GDM at the genetic level and avoid reverse causality and the influence of confounding factors [46], aiming to verify the conclusions of previous epidemiological studies and provide a clearer understanding of the relationship between vitamin D deficiency and GDM.

Moreover, the outcomes of this MR investigation offer Bioinformatics substantiation for prior findings reported by our research team [47]. Our past results indicated a significantly lower proportion of GDM cases in the case group compared to the control group (*p* = 0.015). The findings of this cross-sectional investigation align with the results obtained in our present study. MR provides assistance in cross-validating cross-sectional study findings by addressing the constraints associated with conventional observational epidemiological studies and facilitating causal inference. This method compensates for the limitations of conventional epidemiological study designs and enhances the empirical support for the correlation between vitamin D and GDM.

However, this study still has some limitations. First, a threshold of *p* < 1 × 10^−5^ was used when selecting instrumental variables due to limitations in the GWAS data on vitamin D deficiency, preventing a more stringent threshold. This might introduce some unavoidable confounding factors but provides enough SNPs for analysis. Additionally, the F statistic, which indicates the strength of the instrumental variable for the related exposure, necessitates excluding instrumental variables with an F statistic of less than ten. In the MR study of vitamin D deficiency and GDM, the sample size of the GWAS data for vitamin D deficiency was not provided, making it impossible to calculate the F statistics for the related SNPs. This could result in the inclusion of weak instruments, leading to some bias in the estimates. Secondly, as we used GWAS summarized data, we could not stratify analyses according to demographic characteristics such as sex and age, providing possible future research directions. Additionally, most participants in the GWAS summary data used in the study are of European ancestry. Considering the issue of population stratification, this may introduce some bias in the estimates, and further clinical studies are needed to determine if these results are applicable to other ethnic groups. Moreover, as is the case with many published MR investigations, it is important to acknowledge that despite efforts to detect and eliminate aberrant variance, the potential influence of unobserved pleiotropy on the outcomes cannot be completely dismissed [48]. Furthermore, despite our strict selection criteria, residual confounding factors may still be present, which could cause some bias in the estimates. This study used MR-Egger, weighted median, simple mode, and weighted mode methods for sensitivity analysis and judged the robustness of the causal inference based on the consistency of results across these methods. Nevertheless, in the two MR studies conducted on vitamin D levels and GDM, although the outcomes of each MR analysis aligned with the IVW method, the other four methods showed no evidence of a causal effect. The inconsistency of estimates among different methods indicates that not all genome-wide significant variations of vitamin D levels and vitamin D deficiency in this study were effective instrumental variables [49]. Therefore, the results must be more robust, and robust clinical studies are necessary in order to derive definitive clinical conclusions. Hence, it is necessary to develop more extensive GWAS databases and employ additional analytical techniques or experimental validations to elucidate the connection and mechanisms of action between vitamin D and GDM.

To the best of our knowledge, this paper represents the first utilization of a GWAS dataset to perform a MR analysis, aiming to examine the causal association between vitamin D and GDM. The results provide bioinformatics evidence for the environmental risk factors of GDM, indicating that higher vitamin D levels are a risk factor. For pregnant woman, reducing appropriately vitamin D supplement intake in daily life may be a potential measure for preventing GDM. Additionally, this study offers a possible experimental direction for investigating the molecular mechanisms by which vitamin D affects GDM.

## 5. Conclusions

The findings from the MR study indicate that vitamin D levels are a risk factor for GDM. Additional animal experimentation is required in order to investigate and validate the correlation and influencing mechanisms between vitamin D and GDM. This study explored the causal relationship between vitamin D and gestational diabetes, which could contribute to the prevention of gestational diabetes in the future. It is necessary to conduct longitudinal studies or randomized trials [50] on vitamin D and gestational diabetes in the future to further clarify the causal relationship between vitamin D and gestational diabetes, as well as the detailed mechanisms by which vitamin D exerts its causal effects in gestational diabetes.

## Data Availability

The datasets used and/or analysed during the current study are available from the corresponding author on reasonable request, or can be downloaded from the website https://gwas.mrcieu.ac.uk/ (accessed on 4 February 2024).

## Supplementary Materials

The following supporting information can be downloaded at: https://www.mdpi.com/article/10.3390/nu16172836/s1, Table S1. SNPs used to assess the impact of Vitamin D Deficiency on the risk of Gestational Diabetes Mellitus; Table S2. SNPs used to assess the impact of Vitamin D Levels on the risk of Gestational Diabetes Mellitus; Table S3. SNPs used to assess the impact of Gestational Diabetes Mellitus on Vitamin D Deficiency; Table S4. SNPs used to assess the impact of Gestational Diabetes Mellitus on Vitamin D Levels; Table S5. Results of tests for heterogeneity and pleiotropy of Vitamin D Deficiency and Vitamin D Levels on Gesta-tional Diabetes Mellitus; Table S6. Results of tests for heterogeneity and pleiotropy of Gestational Diabetes Mellitus on Vitamin D Deficiency and Vitamin D Levels; Figure S1. Figure of Leave-One-Out analysis results of Vitamin D Levels and Vitamin D Deficiency on Gestational Dia-betes Mellitus, and Gestational Diabetes Mellitus on Vitamin D Levels and Vitamin D Deficiency; Figure S2. Funnel plot of mendelian randomization analysis results of Vitamin D Levels and Vitamin D Deficiency on Gestational Diabetes Mellitus, and Gestational Diabetes Mellitus on Vitamin D Levels and Vitamin D Deficiency.
