# Supplementary material for: Vitamin D and Gestational Diabetes Mellitus in the IEU OpenGWAS Project: A Two-Sample Bidirectional Mendelian Randomization Study
Source: Nutrients. 2024 Aug 24;16(17):2836. doi: 10.3390/nu16172836 (PMC11397161; doi:10.3390/nu16172836)
Supplement: Supplementary file 1 [file nutrients-16-02836-s001.zip › nutrients-3166758-supplementary.pdf]

nutrients

MDPI

Article

Vitamin D and Gestational Diabetes Mellitus in the IEU OpenGWAS Project: A Two-Sample Bidirectional Mendelian Randomization Study

Yuxuan Bai [1,2,†], Xiaoxiao Wang [1,2,†], Yaxuan Xu [2,3,†], Chang Jiang [1,2], Haoran Liu [1,2], Zixiu Xu [2,3], Jinping Shen [1,2], Xumei Zhang [2,4], Qiang Zhang [1,2] and Yue Du [2,5,*]

1 Department of Occupational and Environmental Health, School of Public Health, Tianjin Medical University, Tianjin 300070, China; baiyuxuan@tmu.edu.cn (Y.B.); wangxiaoxiao@tmu.edu.cn (X.W.); jiangchang@tmu.edu.cn (C.J.); liuhaoran@tmu.edu.cn (H.L.); shenjinping@tmu.edu.cn (J.S.); qiangzhang@tmu.edu.cn (Q.Z.)

2 Key Laboratory of Prevention and Control of Major Diseases in the Population, Ministry of Education, Tianjin Medical University, Tianjin 300070, China; xuyaxuan@tmu.edu.cn (Y.X.); xuzixiu@tmu.edu.cn (Z.X.); zhangxumei@tmu.edu.cn (X.Z.); qiangzhang@tmu.edu.cn (Q.Z.)

3 School of Nursing, Tianjin Medical University, Tianjin 300070, China

4 Department of Nutrition and Food Science, School of Public Health, Tianjin Medical University, Tianjin 300070, China

5 Department of Health Management, School of Public Health, Tianjin Medical University, Tianjin 300070, China

* Correspondence: duyue@tmu.edu.cn

† These authors contributed equally to this work.

Abstract: Background: Gestational diabetes mellitus (GDM) is one of the most prevalent pregnancy problems, and there is still debate over the relationship between vitamin D and GDM. Objectives: Our objective is to investigate the correlation between vitamin D and GDM by employing Mendelian randomization (MR) with summary data obtained from genome-wide association studies (GWAS). Methods: Data on exposures and outcomes, namely vitamin D, vitamin D insufficiency, and GDM, were acquired from the IEU OpenGWAS Project. Bidirectional MR analysis was performed utilizing the inverse variance weighted (IVW) method as the principal analytical approach. The complementary approaches employed in this study encompassed weighted median, simple mode, weighted mode, and MR-Egger regression. A series of sensitivity analysis were conducted in order to assess the reliability of the obtained results. Results: The data were acquired from the IEU OpenGWAS Project. Following the application of the three assumptions of MR, 13 single nucleotide polymorphisms (SNPs) were included in the MR analysis for vitamin D levels and vitamin D deficiency on GDM, and 10 and 26 SNPs were included for GDM on vitamin D levels and deficiency, respectively. The findings from the IVW analysis revealed a significant positive correlation between vitamin D levels and GDM (OR = 1.057, 95% CI: 1.011–1.104, $p = 0.015$). Conversely, a negative correlation was seen between vitamin D deficiency and GDM (OR = 0.979, 95% CI: 0.959–0.999, $p = 0.039$). The results of the reverse MR study revealed no evidence of reverse causation between GDM and vitamin D. The findings from multiple MR approaches were in line with the direction of IVW analysis. Sensitivity analysis revealed no evidence of heterogeneity, pleiotropy, or outliers, suggesting the robustness of the results. Conclusions: There exists a causal association between vitamin D and GDM, whereby vitamin D levels serve as a risk factor for GDM.

Keywords: vitamin D; gestational diabetes mellitus; Mendelian randomization

Supplementary Materials:

Table S1. SNPs used to assess the impact of Vitamin D Deficiency on the risk of Gestational Diabetes Mellitus

SNP	EA	NEA	EAF	Beta	SE	*p*-Value
**rs55813571**	G	A	0.5594	−0.4919	0.1077	4.98×10^{-6}
**rs4689985**	G	A	0.07922	0.9366	0.2113	9.35×10^{-6}
**rs6904437**	G	A	0.2047	0.6125	0.1348	5.49×10^{-6}
**rs79896089**	C	A	0.01591	2.4857	0.5309	2.84×10^{-6}
**rs56027902**	A	G	0.02637	1.7837	0.3968	6.94×10^{-6}
**rs12809078**	T	A	0.07395	1.0252	0.2267	6.09×10^{-6}
**rs17293059**	C	A	0.113	0.8164	0.178	4.50×10^{-6}
**rs191245781**	C	T	0.01268	2.9661	0.6095	1.14×10^{-6}
**rs1618112**	A	C	0.01443	2.5282	0.5481	3.97×10^{-6}
**rs2782984**	A	G	0.8649	−0.7385	0.1626	5.60×10^{-6}
**rs77883004**	T	C	0.05197	1.2902	0.2662	1.25×10^{-6}
**rs970469**	C	T	0.01609	2.4666	0.5213	2.23×10^{-6}
**rs1943958**	G	A	0.02179	2.3009	0.4555	4.38×10^{-7}

Abbreviations: EA, effect allele; EAF, effect allele frequency; SE, standard error; SNP, single-nucleotide polymorphism

Table S2. SNPs used to assess the impact of Vitamin D Levels on the risk of Gestational Diabetes Mellitus

SNP	EA	NEA	Sample Size	EAF	Beta	SE	*p*-Value	R	F-Statistic
**rs10431487**	G	T	1209	0.6012	−0.2378	0.05177	4.36×10^{-6}	−0.13107	21.09927
**rs10488980**	G	A	1209	0.8717	−0.3463	0.07704	6.94×10^{-6}	−0.12832	20.20563
**rs12237106**	T	G	1209	0.6365	0.2542	0.05498	3.77×10^{-6}	0.131918	21.37675
**rs13198611**	C	T	1209	0.8956	0.386	0.08633	7.78×10^{-6}	0.127645	19.99176
**rs139454338**	A	G	1209	0.8718	0.3668	0.07614	1.46×10^{-6}	0.13735	23.20774
**rs1514371**	T	G	1209	0.7728	−0.2691	0.05936	5.80×10^{-6}	−0.12939	20.55131
**rs2228703**	A	G	1209	0.8441	0.3145	0.06658	2.32×10^{-6}	0.134724	22.31278
**rs561614821**	G	A	1209	0.96499	0.644	0.1435	7.21×10^{-6}	0.128111	20.14039
**rs56255035**	C	T	1209	0.98946	−1.2	0.2547	2.46×10^{-6}	−0.13438	22.19753
**rs62314792**	C	T	1209	0.7	−0.2915	0.056	1.94×10^{-7}	−0.14818	27.09574
**rs72797535**	A	G	1209	0.8996	0.3816	0.08115	2.58×10^{-6}	0.134129	22.11259
**rs7604159**	T	C	1209	0.6604	0.2698	0.05315	3.86×10^{-7}	0.144577	25.7678
**rs7757449**	T	C	1209	0.1089	0.3767	0.08473	8.76×10^{-6}	0.126934	19.76591

Abbreviations: EA, effect allele; EAF, effect allele frequency; SE, standard error; SNP, single-nucleotide polymorphism

Table S3. SNPs used to assess the impact of Gestational Diabetes Mellitus on Vitamin D Deficiency

SNP	EA	NEA	Sample Size	EAF	Beta	SE	*p*-Value	R	F-Statistic
**rs10161329**	A	G	373045	0.1638	0.1245	0.0271	4.43×10^{-6}	0.007522	21.10572
**rs10752161**	T	C	373045	0.9048	0.1747	0.0343	3.66×10^{-7}	0.008339	25.94165

**rs111854973**	G	C	373045	0.03057	0.2919	0.06	1.13×10^{-6}	0.007965	23.66823
**rs112512273**	T	C	373045	0.04745	0.2243	0.048	2.99×10^{-6}	0.007651	21.83615
**rs117662621**	T	A	373045	0.07706	−0.187	0.0389	1.54×10^{-6}	−0.00787	23.10915
**rs12673513**	T	C	373045	0.1752	−0.1261	0.0264	1.80×10^{-6}	−0.00782	22.8151
**rs143534034**	T	C	373045	0.02321	0.3313	0.0679	1.06×10^{-6}	0.007988	23.80693
**rs144589137**	A	G	373045	0.03051	−0.2847	0.0598	1.95×10^{-6}	−0.00779	22.66588
**rs145584736**	A	G	373045	0.01683	0.3931	0.0785	5.47×10^{-7}	0.008199	25.07649
**rs150558253**	G	C	373045	0.01936	0.3286	0.0726	6.02×10^{-6}	0.00741	20.48622
**rs188282457**	C	A	373045	0.02042	−0.3336	0.0727	4.49×10^{-6}	−0.00751	21.05636
**rs1889384**	A	G	373045	0.2509	−0.105	0.0233	6.47×10^{-6}	−0.00738	20.30798
**rs191615758**	A	G	373045	0.0868	0.1703	0.0359	2.04×10^{-6}	0.007767	22.503
**rs2076281**	G	A	373045	0.09349	0.1534	0.0346	9.53×10^{-6}	0.007259	19.65615
**rs2466294**	G	C	373045	0.6148	−0.1088	0.0207	1.53×10^{-7}	−0.00861	27.62594
**rs34226389**	C	T	373045	0.03757	0.2397	0.0533	6.94×10^{-6}	0.007363	20.22468
**rs3770704**	G	A	373045	0.03263	0.2477	0.0557	8.76×10^{-6}	0.007281	19.77614
**rs56322010**	A	G	373045	0.1743	0.1182	0.0265	8.43×10^{-6}	0.007303	19.89497
**rs59649116**	A	C	373045	0.2158	−0.1412	0.0248	1.31×10^{-8}	−0.00932	32.41649
**rs62331897**	C	T	373045	0.05009	0.2059	0.0463	8.66×10^{-6}	0.007281	19.77656
**rs6449108**	T	C	373045	0.4601	−0.0897	0.0202	8.57×10^{-6}	−0.00727	19.71888
**rs7602543**	A	C	373045	0.1248	−0.1401	0.0305	4.30×10^{-6}	−0.00752	21.09972
**rs7621117**	G	A	373045	0.8028	−0.1165	0.0253	4.14×10^{-6}	−0.00754	21.20366
**rs77308130**	A	G	373045	0.01698	0.3616	0.0806	7.30×10^{-6}	0.007345	20.12736
**rs78868683**	C	G	373045	0.02628	0.2969	0.0667	8.63×10^{-6}	0.007288	19.81384
**rs79896666**	C	A	373045	0.07847	−0.1722	0.0375	4.28×10^{-6}	−0.00752	21.08646

Abbreviations: EA, effect allele; EAF, effect allele frequency; SE, standard error; SNP, single-nucleotide polymorphism

Table S4. SNPs used to assess the impact of Gestational Diabetes Mellitus on Vitamin D Levels

SNP	EA	NEA	Sample Size	EAF	Beta	SE	*p*-Value	R	F-Statistic
**rs10161329**	A	G	373045	0.1638	0.1245	0.0271	4.43×10^{-6}	0.007522	21.10572
**rs12673513**	T	C	373045	0.1752	−0.1261	0.0264	1.80×10^{-6}	−0.00782	22.8151
**rs1889384**	A	G	373045	0.2509	−0.105	0.0233	6.47×10^{-6}	−0.00738	20.30798
**rs2076281**	G	A	373045	0.09349	0.1534	0.0346	9.53×10^{-6}	0.007259	19.65615
**rs2466294**	G	C	373045	0.6148	−0.1088	0.0207	1.53×10^{-7}	−0.00861	27.62594
**rs56322010**	A	G	373045	0.1743	0.1182	0.0265	8.43×10^{-6}	0.007303	19.89497
**rs6449108**	T	C	373045	0.4601	−0.0897	0.0202	8.57×10^{-6}	−0.00727	19.71888
**rs7602543**	A	C	373045	0.1248	−0.1401	0.0305	4.30×10^{-6}	−0.00752	21.09972
**rs7621117**	G	A	373045	0.8028	−0.1165	0.0253	4.14×10^{-6}	−0.00754	21.20366
**rs78868683**	C	G	373045	0.02628	0.2969	0.0667	8.63×10^{-6}	0.007288	19.81384

Abbreviations: EA, effect allele; EAF, effect allele frequency; SE, standard error; SNP, single-nucleotide polymorphism

Table S5. Results of tests for heterogeneity and pleiotropy of Vitamin D Deficiency and Vitamin D Levels on Gestational Diabetes Mellitus

Exposure Factor	Outcome Factor	IVW			MR-Egger Intercept		
		Q	*df*	*p*-Value	Intercept	SE	*p*-Value
Vitamin D deficiency	GDM	13.090	12	0.363	−0.010	0.021	0.661
Vitamin D	GDM	6.374	12	0.896	0.010	0.028	0.725

Abbreviations: IVW, Inverse variance weighted; MR, Mendelian randomization; SE, standard error; GDM, gestational diabetes mellitus

Table S6. Results of tests for heterogeneity and pleiotropy of Gestational Diabetes Mellitus on Vitamin D Deficiency and Vitamin D Levels

Exposure Factor	Outcome Factor	IVW			MR-Egger Intercept		
		Q	*df*	*p*-Value	Intercept	SE	*p*-Value
GDM	Vitamin D Deficiency	21.272	25	0.677	0.076	0.091	0.414
GDM	Vitamin D	3.149	9	0.958	0.014	0.053	0.806

Abbreviations: IVW, Inverse variance weighted; MR, Mendelian randomization; SE, standard error; GDM, gestational diabetes mellitus

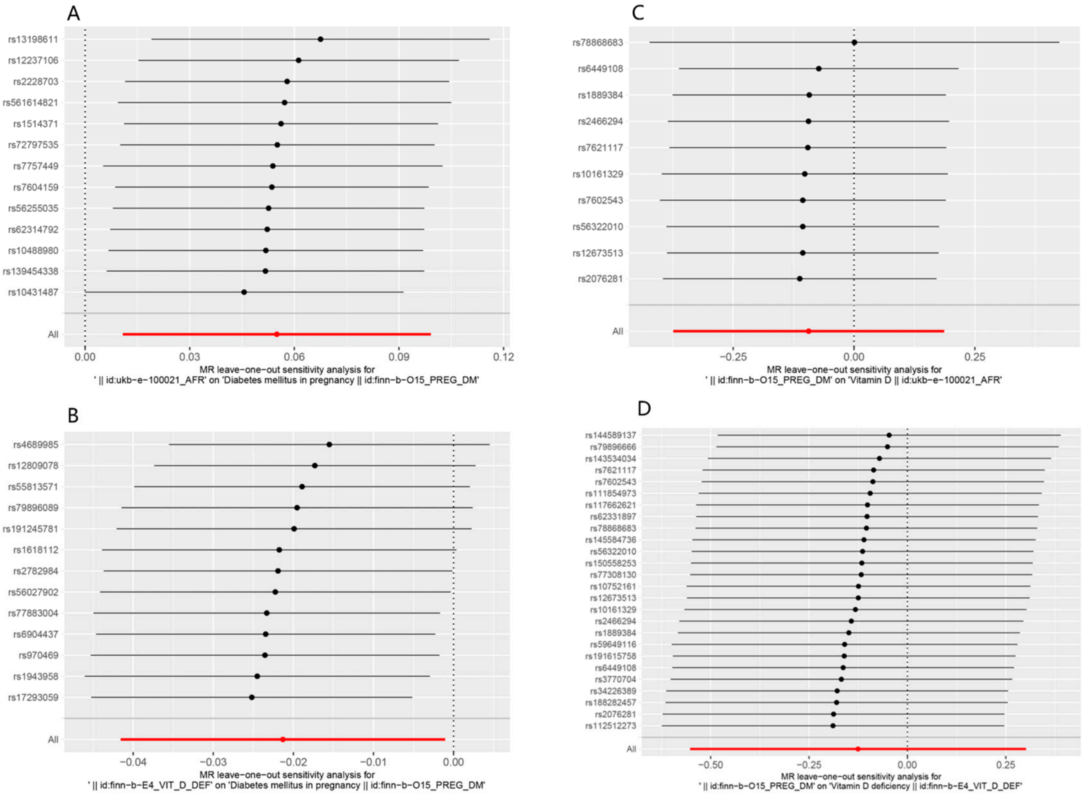

Figure S1. Figure of Leave-One-Out analysis results of Vitamin D Levels and Vitamin D Deficiency on Gestational Diabetes Mellitus, and Gestational Diabetes Mellitus on Vitamin D Levels and Vitamin D Deficiency. Figures (A–D) respectively show the leave-one-out analysis results of (A) Vitamin D levels and Gestational Diabetes Mellitus, (B) Vitamin D Deficiency and Gestational Diabetes Mellitus, (C) Gestational Diabetes Mellitus and Vitamin D levels, and (D) Gestational Diabetes Mellitus and Vitamin D Deficiency.

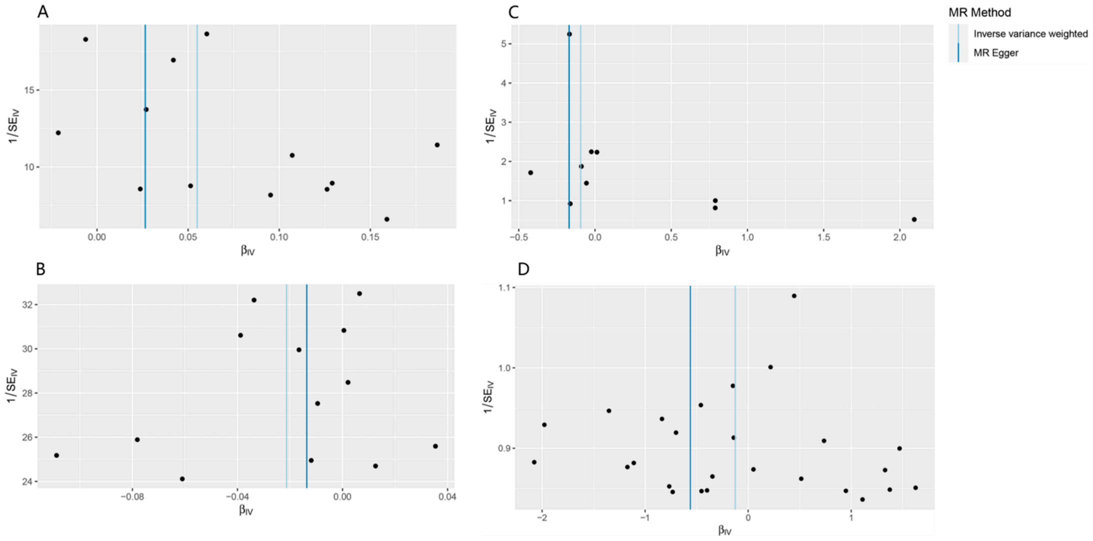

Figure S2. Funnel plot of mendelian randomization analysis results of Vitamin D Levels and Vitamin D Deficiency on Gestational Diabetes Mellitus, and Gestational Diabetes Mellitus on Vitamin D Levels and Vitamin D Deficiency. Figures (A–D) respectively show the funnel plots of Mendelian randomization analysis results of (A) Vitamin D levels and

Gestational Diabetes Mellitus, (B) Vitamin D Deficiency and Gestational Diabetes Mellitus, (C) Gestational Diabetes Mellitus and Vitamin D levels, and (D) Gestational Diabetes Mellitus and Vitamin D Deficiency.
